# Actinidin in Green and SunGold Kiwifruit Improves Digestion of Alternative Proteins—An In Vitro Investigation

**DOI:** 10.3390/foods11182739

**Published:** 2022-09-06

**Authors:** Lovedeep Kaur, Boning Mao, Justine Bailly, Olawunmi Oladeji, Paul Blatchford, Warren C. McNabb

**Affiliations:** 1 School of Food and Advanced Technology, Massey University, Palmerston North 4442, New Zealand; 2 Riddet Institute, Massey University, Palmerston North 4442, New Zealand; 3 Institut Agro—Agrocampus Ouest, 35042 Rennes, France; 4 Zespri International Limited, Mt. Maunganui 3116, New Zealand

**Keywords:** actinidin, green kiwifruit, gold kiwifruit, in vitro digestion, alternative proteins, SDS-PAGE

## Abstract

Both Hayward (green) and SunGold (gold) kiwifruit varieties contain a proteolytic enzyme, actinidin, that has been reported to enhance the upper tract digestion of animal proteins. Unlike the other gold varieties, which do not contain any actinidin, the SunGold variety contains significantly higher actinidin activity, but its activity is still much lower than that present in the green (Hayward) fruit. The objective of this study was to determine the effectiveness of actinidin in Hayward and SunGold kiwifruit in digesting alternative proteins, including pea protein, almonds, tofu, and quinoa. The protein sources were digested using a three-stage in vitro oral-gastro-small intestinal digestion model. The findings showed that both kiwifruit extracts enhanced the breakdown (observed through SDS-PAGE) for all the studied protein sources, particularly during gastric digestion, possibly due to higher actinidin activity at gastric pH. The increase in the rate of protein breakdown was probably due to the broader specificity of actinidin compared to pepsin. For many protein sources, most of the intact proteins disappeared within the first few minutes of gastric digestion with added kiwifruit extract. Green kiwifruit extract, due to its higher actinidin activity, had a higher effect on protein breakdown than the SunGold extract. However, for some proteins and under certain digestion conditions, SunGold extract resulted in higher protein breakdown. The latter, in the absence of any digestive enzymes, also led to some protein breakdown during the small intestinal digestion phase, which was not the case for the green kiwifruit extract. The green kiwifruit extract led to the greater breakdown of polypeptide chains of Pru-du 6, a major allergen in almonds. The results, for the first time, suggest that both Hayward and SunGold kiwifruit can lead to improved breakdown and digestion of alternative proteins when consumed as part of a meal; and therefore, have the potential to be used as a digestive aid in population groups looking to achieve faster and greater protein digestion such as athletes, elderly and people with the impaired digestive system.

## 1. Introduction

Alternative sources of proteins are gaining popularity as they are seen as an environmentally friendly answer to the growing global demand for protein foods. However, there is insufficient knowledge on how these proteins are digested in the human digestive tract and how their digestion may be influenced by other components of the diet. Enhancing the digestion of dietary proteins through means of dietary intervention may reduce the potential negative effects of consuming high protein diets, particularly those that are slower to digest [1,2,3].

Owing to the presence of a proteolytic enzyme, actinidin, green kiwifruit (*Actinidia deliciosa* var. ‘Hayward’) has been shown to enhance the digestion of a variety of animal food proteins over and above that of the digestive enzymes alone [1,4,5,6,7,8]. Actinidin has also been reported to hydrolyse gluten proteins under simulated gastric conditions [9]. This enzyme showed a greater degree of hydrolysis of gliadin when compared with other exogenous enzymes, such as papain and bromelain. The greater effect of actinidin in the gastric phase than in the small intestinal digestion phase has been linked with its near-optimal pH for actinidin activity, which has been reported to be 4 when using food proteins as substrates [4,10].

The broad specificity of actinidin compared to digestive enzymes, particularly pepsin, results in hydrolysis of a wide range of peptide bonds, thus exposing new sites for further hydrolysis by the digestive enzymes [1]. Therefore, actinidin has been suggested to compensate for the loss of natural digestive ability in some population groups such as the elderly [2]. It has also been reported to speed up gastric emptying (as observed in rats and with Hayward fruit, thus making the digesta more quickly available to the absorptive systems in the small intestine [6]. The rates of release of amino acids, particularly during gastric digestion of proteins, may have important implications for modulating amino acid delivery to cells. Faster amino acid delivery to cells is relevant for individuals interested in promoting muscle protein synthesis (e.g., athletes and the elderly) [11,12]. Faster amino acid absorption may also increase satiation (termination of eating) as energy from derived protein is more satiating than energy derived from carbohydrates and fat [13]. A recent study has reported that the consumption of green kiwifruit along with beef leads to a faster increase in peripheral plasma essential amino acid concentrations [14].

Actinidin is a cysteine protease like bromelain, ficain or papain [15]. It has been reported to strongly hydrolyse the amide and ester bonds at the carboxyl side of a lysine residue [16,17,18,19]. It has a broader active pH range (pH 3 to 10) than other sulfhydryl proteases including papain, ficain and bromelain; has an optimum temperature of around 40 °C and has a lower inactivation temperature of 60 °C [2,15,17,20,21,22,23]. SunGold kiwifruit variety contains high amounts of vitamins C and E [24]. Unlike the other gold varieties which do not contain any actinidin, this variety contains significantly higher actinidin activity, but its activity is still much lower than that present in the green (Hayward) fruit [25]. It was hypothesised that despite having lower actinidin activity, SunGold kiwifruit would still be able to hydrolyse proteins over and above that of the digestive enzymes alone, possibly leading to enhancement of the rate of protein digestion. We also hypothesised that the above-discussed effects of actinidin present in green kiwifruit on the digestion of animal protein sources would be replicated with alternative protein sources. The results of the study may be useful in understanding the role of kiwifruit consumption as part of an alternative protein-based meal. Thus, the project’s main aim was to determine the effects of actinidin in green (*Actinidia deliciosa* var. ‘Hayward’) and SunGold (*Actinidia chinensis* var. ‘Zesy002’) kiwifruit on the digestion of selected alternative protein sources, using an in vitro digestion model. Two established complementary analytical approaches: sodium dodecyl sulfate-polyacrylamide gel electrophoresis (SDS-PAGE), to develop an understanding of the soluble proteins present in food and their disappearance during digestion, and free amino nitrogen determination (ninhydrin-reactive free amino N), which is a quantitative measure of the number of peptide bonds hydrolysed, were used. The SDS-PAGE results are most useful as they show the disappearance of intact proteins and thus indicate the start of breakdown by hydrolysis. While the appearance of free amino N is a useful quantitative indicator, in many cases, the precision of the method is not enough to reveal some of the quite small changes that will occur during initial protein breakdown.

## 2. Materials and Methods

### 2.1. Materials

Hayward and SunGold kiwifruit used in this study were supplied by ZESPRI (New Zealand). The fruit was stored at 4 °C until extraction was carried out. 

The four foods/proteins used in the study were tofu (firm style, Bean Supreme, NZ), pea protein isolate (PPI, Davis Food Ingredients, NZ), raw almonds (Mother Earth, NZ), and quinoa (Pams, NZ). All the foods were bought from a local market. Pepsin (porcine gastric mucosa; 800–2500 units/mg protein), pancreatin (hog pancreas; 4 × USP) and ninhydrin reagent were purchased from Sigma-Aldrich Pty. Ltd.(Burlington, MA, USA). All other chemicals and reagents used in the study were of analytical grade. 

### 2.2. Preparation of Kiwifruit Extract and Determination of Enzyme Activity (U/mL of Extract or U/g Fresh Fruit)

Kiwifruit extract (KE) was prepared and analysed for enzyme activity using the method of Boland and Hardman (1972) as previously described by [4] Kaur et al. (2010a) by using N-α-CBZ-lys-*p*-nitrophenol hydrochloride (Z-lys-*p*Np, Sigma, Sigma-Aldrich Pty Ltd. (Burlington, MA, USA)) as a substrate. In brief, kiwifruit was peeled and pressed in a Cheese Press (SMC Pneumatics, New Zealand) through a muslin cloth. The filtrate obtained was centrifuged (Sorvall RC 6 Plus Centrifuge, Thermo Scientific, Auckland, New Zealand) at 13,089× *g* for 30 min at 0 °C. The supernatant was immediately stored at 4 °C for enzyme activity measurement.

### 2.3. Sample Preparation

Quinoa was cooked by adding half a cup of quinoa into one cup of water and cooked in a rice cooker for 25 min. It was then cooled to 20 °C, vacuum-sealed and used (within an hour) for digestion experiments. For pea protein isolate, one (1) gram of PPI was suspended in five (5) grams of Milli-Q water and stirred using a magnetic stirrer until a homogenous paste was obtained. All solid foods were crushed (to approximately 2 mm) using a pestle and mortar before using them for in vitro digestion experiments.

Kiwifruit extracts and food proteins were analysed for nitrogen content using the AOAC Kjeldahl method [26].

### 2.4. In Vitro Digestibility of Proteins

A 3-stage model system was used to represent oral, stomach and small intestinal digestion of proteins based on the methods of [4,5,27,28] Minekus et al. (2014), Kaur et al. (2010 a, b) and Chian et al. (2019) with some modifications as described below. Foods were crushed (except PPI), to mimic chewing, immediately before adding into the digestion reactors. Kiwifruit extracts were prepared as previously described and added to the digestion mix at the beginning of gastric digestion. 

The amount of kiwifruit extract added was calculated based on one serving of food/protein (different for each protein source) to extract prepared from one serve (2 fruits) of kiwifruit (Table 1). In the text, extracts prepared from SunGold, and Hayward kiwifruits are referred to as ‘Y’ and ‘K’. 

Digestion regimes included two controls and two treatments for each protein source:

(1) CONTROL 1: no enzymes added but incubated in simulated oral juice for 2 min then simulated gastric juice at pH 3 for 2 or 5 or 10 or 30 or 60 min then simulated small intestinal juice at pH 7 for 10, or 60 or 120 min (referred to as ‘PC, QC, AC or TC for pea, quinoa, almonds and tofu, respectively)
(2) CONTROL 2: a second control with alpha-amylase (simulated oral juice for 2 min), pepsin (simulated gastric juice at pH 3 for 2 or 5 or 10 or 30 or 60 min) and pancreatin solution (simulated small intestinal juice at pH 7 for 10, or 60 or 120 min), but no kiwifruit extract (referred to as ‘PE, QE, AE or TE for pea, quinoa, almonds and tofu, respectively).
(3) TREATMENT 1: alpha-amylase (simulated oral juice for 2 min), pepsin (simulated gastric juice at pH 3 for 2 or 5 or 10 or 30 or 60 min) and pancreatin solution (simulated small intestinal juice at pH 7 for 10, or 60 or 120 min) with kiwifruit extract added at the beginning of the gastric digestion (referred to as ‘PKE/PYE, QKE/QYE, AKE/AYE or TKE/TYE for Hayward/gold kiwifruit extracts containing pea, quinoa, almonds and tofu digests, respectively).
(4) TREATMENT 2: no digestive enzymes were added but incubated with kiwifruit extract added at the beginning of the gastric digestion. The samples were digestion in simulated oral juice for 2 min then simulated gastric juice at pH 3 for 2 or 5 or 10 or 30 or 60 min then simulated small intestinal juice at pH 7 for 10, or 60 or 120 min (referred to as ‘PK/PY, QK/QY, AK/AY or TK/TY for green/gold kiwifruit extracts containing pea, quinoa, almonds and tofu digests, respectively).

#### 2.4.1. Analyses Performed on Digests

Aliquots were taken after designated digestion times and pepsin or pancreatin inhibitors were added immediately [28] to stop the digestive enzyme action, followed by homogenisation and storing at −20 °C until analysed.

##### Tricine SDS-PAGE

The homogenised digests were examined for protein breakdown using reduced-Tricine- SDS-polyacrylamide gel electrophoresis as described by [28] Chian et al. (2019). The digests were mixed with tricine sample buffer and then loaded onto the gel at a protein concentration of 1 mg/mL. Gels (16.5% gradient Tricine gels, Criterion^TM^ Precast Gel, Bio-Rad Laboratories) were run using a Criterion^TM^ cell (Bio-Rad Laboratories Pty. Ltd., New Zealand) at a constant voltage of 125 V and were stained before scanning using a gel scanning densitometer (Molecular Imager Gel Doc XR, Bio-Rad Laboratories Pty. Ltd., New Zealand). Images were analysed using the Image Lab^TM^ software (version 6.0.1 build 34, Bio-Rad Laboratories, NZ).

Major components were identified in control protein digests based on their molecular weights. 

##### Ninhydrin-Reactive Free Amino N (%)

Digests were thawed and centrifuged at 10,000× *g* for 20 min at 4 ℃ using a high-speed refrigerated centrifuge (CR 22 GII, Himac, Hitachi Koki Co., Ltd., Tokyo, Japan). The supernatant was then filtered through a 0.45 μm PVDF filter (Millex ^®^) before analysing for ninhydrin reactive amino N (%) using the method described by [29] Moore (1968) using ninhydrin reagent [28].

Because the kiwifruit extract and the digestive enzymes all contain proteins that can self-digest as well as non-protein amino compounds, the results for this were used to correct values obtained with food proteins to obtain a net difference.

##### Soluble Nitrogen (%)

The kiwifruit extracts and clear digest supernatants were also analysed for soluble nitrogen (%) using the AOAC Kjeldahl method [26].

### 2.5. Statistical Analysis

The results are presented as a means ± standard deviation (SD) of triplicate observations. Data were analysed using Minitab 19.1.1 statistical software (Minitab Inc., State College, PA, USA). One-way analysis of variance (ANOVA) followed by Tukey’s Pairwise Comparisons at a 5% confidence level was used to analyse the data (*p* < 0.05). Densitograms are not shown as the hydrolysis of proteins during digestion by actinidin and/digestive enzymes is quite pronounced and clear in the presented SDS-PAGE gel electrophoretograms.

## 3. Results and Discussion

### 3.1. Nitrogen/Protein Contents of Foods and Enzyme Activity of Kiwifruit Extracts

The nitrogen and protein contents of the foods are presented in Table 1. As expected, the highest protein content was observed for PPI and almonds whereas quinoa had the lowest. 

The average enzyme activity for SunGold kiwifruit extract used in the study was about ~28% of the enzyme activity of green kiwifruit (Hayward) extract. This agrees with the previously reported values. It is also noticeable that the amount of extract obtained from SunGold kiwifruit (50 mL/kiwifruit on average was higher than that obtained from Hayward (30 mL/kiwifruit on average) fruit. This could be due to the presence of higher water and lower amounts of total fibre (particularly insoluble fibre) in the former (Sivakumaran et al., 2018). This suggests the total enzyme activity of extract prepared from one SunGold kiwifruit to be about half of that observed for Hayward kiwifruit (Table 1). 

### 3.2. Pea Protein Isolate (PPI)

#### 3.2.1. SDS-PAGE

**Figure 1:**
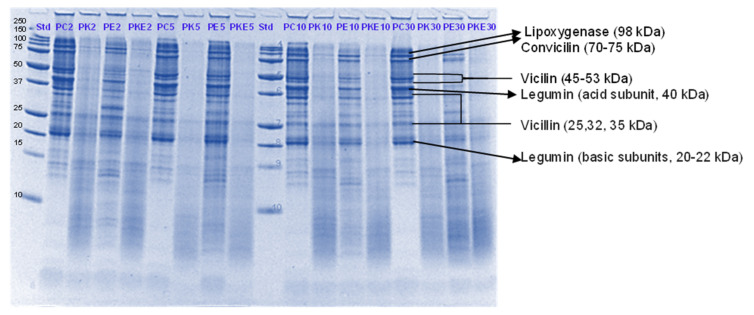
Tricine SDS-PAGE electrophoretogram of pea protein isolate after 2, 5, 10, and 30 min of simulated oral-gastric digestion in the presence or absence of **Hayward (K)** kiwifruit extract. PC, Pea protein (P) control without any digestive enzymes; PK, with added green kiwifruit extract but no digestive enzymes; PE, with digestive enzymes but no added kiwifruit extract; PKE, with both digestive enzymes and green kiwifruit extract. Numbers 2, 5, 10, and 30 are gastric digestion times following 2 min of oral digestion. Bands (1–10) in the molecular weight standard lane correspond to molecular weights 250, 150, 100, 75, 50, 37, 25, 20, 15 and 10 kDa.

**Figure 2:**
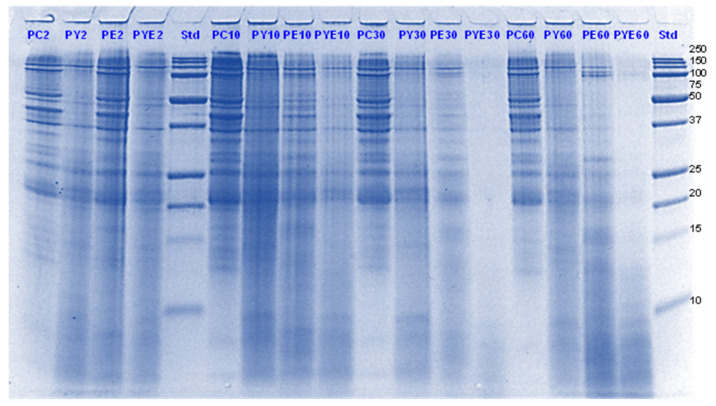
Tricine SDS-PAGE electrophoretogram of pea protein isolate after 2, 10, 30 and 60 min of simulated oral-gastric digestion in the presence or absence of **SunGold (Y)** kiwifruit extract. PC, Pea protein (P) control without any digestive enzymes; PY, with added gold kiwifruit extract but no digestive enzymes; PE, with digestive enzymes but no added kiwifruit extract; PYE, with both digestive enzymes and gold kiwifruit extract. Numbers 2, 10, 30 and 60 are gastric digestion times following 2 min of oral digestion. Bands (1–10) in the molecular weight standard lane correspond to molecular weights 250, 150, 100, 75, 50, 37, 25, 20, 15 and 10 kDa.

**Figure 3:**
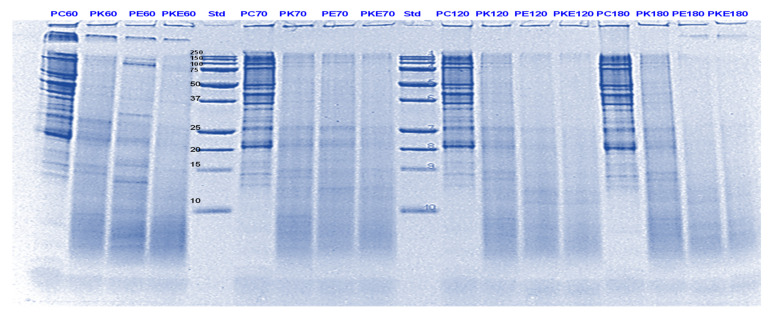
Tricine SDS-PAGE electrophoretogram of pea protein isolate after 60, 70, 120 and 180 min of simulated oral-gastric-small intestinal digestion in the presence or absence of **Hayward (K)** kiwifruit extract. PC, Pea protein (P) control without any digestive enzymes; PK, with added kiwifruit extract but no digestive enzymes; PE, with digestive enzymes but no added kiwifruit extract; PKE, with both digestive enzymes and green kiwifruit extract. The number 60 is the gastric digestion time following 2 min of oral digestion. Numbers 70, 120 and 180 are the total digestion times in oral-gastro-small intestinal digestion (including 60 min of gastric digestion). Bands (1–10) in the molecular weight standard lane correspond to molecular weights 250, 150, 100, 75, 50, 37, 25, 20, 15 and 10 kDa.

**Figure 4:**
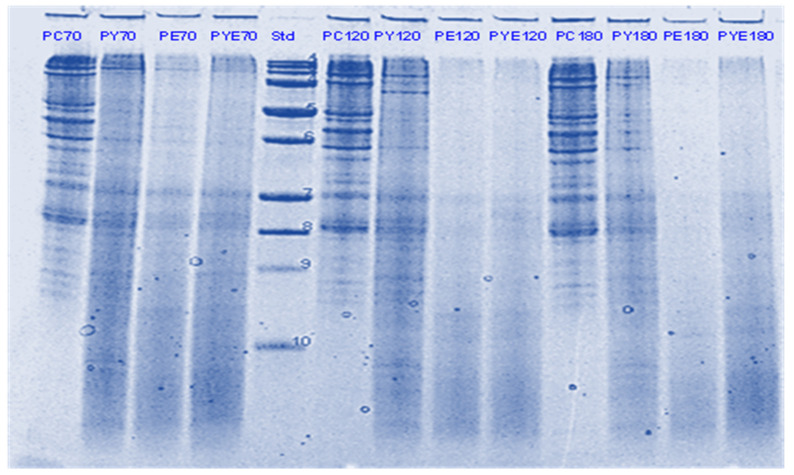
Tricine SDS-PAGE electrophoretogram of pea protein isolate after 70, 120 and 180 min of simulated oral-gastric-small intestinal digestion in the presence or absence of **SunGold (Y)** kiwifruit extract. PC, Pea protein (P) control without any digestive enzymes; PY, with added kiwifruit extract but no digestive enzymes; PE, with digestive enzymes but no added kiwifruit extract; PYE, with both digestive enzymes and gold kiwifruit extract. Numbers 70, 120 and 180 are the total digestion times in oral-gastro-small intestinal digestion (including 60 min of gastric digestion). Bands (1–10) in the molecular weight standard lane correspond to molecular weights 250, 150, 100, 75, 50, 37, 25, 20, 15 and 10 kDa.

The digestibility of pea proteins has been reported to be lower than that of soy and animal proteins due to their compact structure [3]. There are two main classes of proteins present in peas- globulins and albumins. Globulins represent 55–65% of the total protein present in pea seeds and are composed of legumin (11S) and vicilin (7S) [30,31]. The globulin fraction is known to be more digestible than the albumin fraction. The latter is comprised of lipoxygenase, lectins and other antinutritional components. Four major identified components (based on their molecular weights) in the control PPI digest are lipoxygenase (98 kDa), convicilin (70–75 kDa), vicilin (45–53, 32, 25 and 35 kDa), legumin (acid subunit, 40 kDa and basic subunits, 20–22 kDa) and lectin subunits (17 kDa) [32] (Figure 1, Figure 2, Figure 3 and Figure 4).

Convicilin subunits and to some extent lipoxygenase, legumin (basic subunits, 20 kDa) and vicilin (25 kDa) were found to be more resistant to gastric digestion than legumin acidic subunits and other vicilin subunits as the bands corresponding to the latter nearly disappeared after 60 min of gastric digestion in the samples hydrolysed by pepsin alone. This shows that convicilin and other resistant proteins have a structure (amino acid composition) that is quite compact and stable even at acidic pH (3) to resist the cleavage by pepsin. Convicilin contains one cysteine residue contrary to the majority of 7S globulins that could have contributed to more stability of its structure [33]. It is important to mention that the pH used in this study was 3 which is higher than the optimum pH of 1.2 for pepsin [34]. Both Hayward and SunGold kiwifruit extracts alone (with no added pepsin) were also able to almost completely digest convicilin subunits, with their disappearance after the first few seconds of gastric digestion. Actinidin has a broader specificity than pepsin [1], which is possibly the reason that green or SunGold extract (with or without any added pepsin) digested all the other major proteins present in PPI significantly during the gastric incubation phase over and above than pepsin alone, as seen by near-disappearance of their corresponding bands (Figure 1, Figure 2, Figure 3 and Figure 4). This shows that actinidin in kiwifruit when consumed as part of a protein-based meal can lead to enhancement of the rate of pea protein digestion in the gastric phase. It is important to note that actinidin in Hayward led to faster and greater hydrolysis of pea proteins in the absence of pepsin than actinidin in SunGold whereas the effect of actinidin in the presence of pepsin was greater for SunGold than green extract. Greater breakdown of proteins by green kiwifruit extract was expected as it has greater actinidin activity than the Hayward extract. However, lower protein breakdown by green than gold kiwifruit in the presence of pepsin after 30 and 60 min of digestion might be due to the presence of higher dietary fibre in green than gold kiwifruit, which has been reported to impact the mass transfer (diffusion coefficient) of the molecules, including sugars, peptides and enzymes; and the enzymatic reactions during digestion [16].

The results also show that actinidin alone is capable of hydrolysing the majority of the intact pea proteins but is not able to hydrolyse the peptides produced during hydrolysis of the intact pea protein. This is the reason for greater protein hydrolysis when both actinidin and pepsin are present. The hydrolysis of the peptide bonds that are either inaccessible to pepsin or pepsin has no specificity for actinidin possibly opened up the pea proteins’ structure and exposed new sites for pepsin action [1], thereby enhancing their digestion over and above that by pepsin alone.

Pepsin has been known to be the main enzyme responsible for the cleavage of folded native proteins whereas the role of pancreatic enzymes involves the generation of single amino acids or very short peptides for efficient uptake by the intestinal mucosa [34]. In this study, it was quite clear that the proteins and peptides which were not digested by pepsin or actinidin were digested to a significant extent by the pancreatic enzymes. However, throughout, and at the end of gastro-small intestinal digestion (Figure 3 and Figure 4), PPI digested in the presence of both green kiwifruit extract and digestive enzymes (PKE) showed greater protein breakdown than that digested in the presence of digestive enzymes alone (PE) as seen from a greater decrease in intensity of the bands corresponding to all the proteins and peptides produced during the gastric digestion phase. Minor hydrolysis of proteins and peptides released during gastric digestion was also seen in the kiwifruit extract-only samples (with no added digestive enzymes). The observed major effect of actinidin during gastric digestion whether alone or in the presence of digestive enzymes, suggests its possible role in the digestion of proteins for people with impaired gastric digestion systems. Proteolytic effects of the pancreatic enzymes are largely due to their multiple specificities, leading to more complete hydrolysis of the proteins and peptides than actinidin alone [34]. Some proteins and peptides were digested in the presence of digestive enzymes but in the absence of SunGold kiwifruit extract seemed to have a better breakdown than when SunGold kiwifruit extract was added along with digestive enzymes. Similar results have been reported by [2] Kaur and Boland (2013) for soy proteins but with Hayward kiwifruit extract. This could be due to the presence of non-protein components in the SunGold extract, which might have impacted the protein breakdown. Another possibility is the partial breakdown of the digestive enzymes by actinidin present in SunGold kiwifruit. 

The results suggest that actinidin present in green or SunGold kiwifruit (with no added pepsin) is capable of acting efficiently over and above that of pepsin in hydrolysing peptide bonds of native pea proteins and can cleave peptide bonds that are not digested by pepsin in an acidic environment. This is highly important, particularly for population groups with impaired digestive systems, such as people with partial gastrectomy or the elderly. Pepsin hydrolysis has been reported to be not necessary for survival and that a combination of the pancreatic enzymes can be sufficient when acting together for our survival [35]. However, consuming actinidin (in green or gold kiwifruit) as part of a meal may be able to perform similar functioning as pepsin and may make up for the loss in pepsin activity. This study showed that the rate of gastric digestion was considerably enhanced in the presence of actinidin along with pepsin, showing the benefits of consuming actinidin (kiwifruit) for the normal population too.

#### 3.2.2. Free Amino N (%) and Soluble Nitrogen (%)

SDS-PAGE provided insights on the proteolytic susceptibility of individual proteins present in pea protein isolate, but it could not detect amino acids and very short peptides (MW < 2–3 kDa) generated from digestion. Thus, a quantitative measurement of amino groups produced during digestion of PPI was performed using the ninhydrin method. 

**Figure 5:**
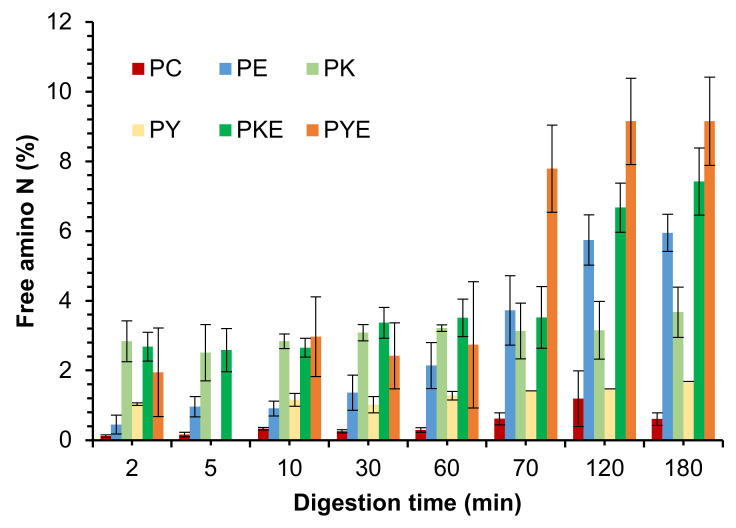
Free amino N (%) release during simulated gastro-small intestinal digestion of pea protein isolate in the presence or absence of Hayward or SunGold kiwifruit extract. PC, Pea protein (P) control without any digestive enzymes; PK/PY, with added kiwifruit extract but no digestive enzymes; PE, with digestive enzymes but no added kiwifruit extract; PKE/PYE, with both digestive enzymes and green/gold kiwifruit extract. The number 60 is the gastric digestion time following 2 min of oral digestion. Numbers 70, 120 and 180 are the total digestion times in oral-gastro-small intestinal digestion (including 60 min of gastric digestion).

Pea protein is commonly used in the manufacturing of meat analogues [3,36]. Therefore, it is relevant to mention that the overall free amino N (%) values released during digestion of PPI were lower than that usually observed for meat [2,22,28]. This agrees with previous studies that suggested lower digestibility of pea proteins than meat [3]. During the gastric digestion phase, all the green kiwifruit extract containing PPI digests showed higher free amino N values than the protein digested in the presence of digestive enzymes alone (Figure 5). This shows an improvement in the rate of protein breakdown and peptide release during the gastric phase. However, for the SunGold kiwifruit containing digests, this was true for the initial phase (first 10 min) of gastric digestion only. Improved protein breakdown and peptide release in the presence of Hayward kiwifruit extract (no added digestive enzymes) during the gastric phase was consistent with improved protein solubility in the presence of kiwifruit extract (data not presented).

The pancreatic endo- and exopeptidases have been reported to efficiently reduce the size of the peptides produced during the gastric digestion phase, which is the reason for a large increase in the free amino N (%) for all the samples during the small intestinal digestion phase. Amino acid and peptide transporters of the small intestine have been found to only take up peptides of a size of four amino acids or smaller, and even the size of four amino acids is less efficiently absorbed compared to shorter peptides making the subsequent cleavage of small peptides generated by pepsin into very short peptides or single amino acids very important [34]. At the end of gastro-small intestinal digestion, the PPI digests containing both digestive enzymes and kiwifruit extract seemed to exhibit higher free amino N release compared to the digestive enzymes containing digests. However, this was more pronounced in the SunGold kiwifruit extract containing digests. As discussed in Section 3.2.1, the reason could be the opening up of the structure of the proteins through hydrolysis of hidden bonds by actinidin for further action by the digestive enzymes and may also be attributed to the differences in the composition of SunGold and Hayward extracts. The latter contains higher dietary fibre which might have impacted the protein/peptide hydrolysis not only during gastric but also during the small intestinal digestion of PPI. The composition of the food matrix has been reported to impact the enzymatic hydrolysis of the proteins [22,36,37].

### 3.3. Tofu

#### 3.3.1. SDS-PAGE

**Figure 6:**
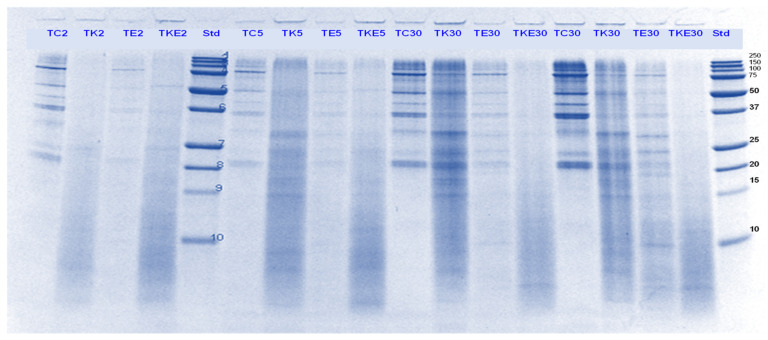
Tricine SDS-PAGE electrophoretogram of tofu (T) digest after 2, 5, 10, and 30 min of simulated oral-gastric digestion in the presence or absence of **Hayward (K)** kiwifruit extract. TC, Tofu (T) control without any digestive enzymes; TK, with added kiwifruit extract but no digestive enzymes; TE, with digestive enzymes but no added kiwifruit extract; TKE, with both digestive enzymes and green kiwifruit extract. Numbers 2, 5, 10, and 30 are the gastric digestion time following 2 min of oral digestion. Bands (1–10) in the molecular weight standard lane correspond to molecular weights 250, 150, 100, 75, 50, 37, 25, 20, 15 and 10 kDa.

**Figure 7:**
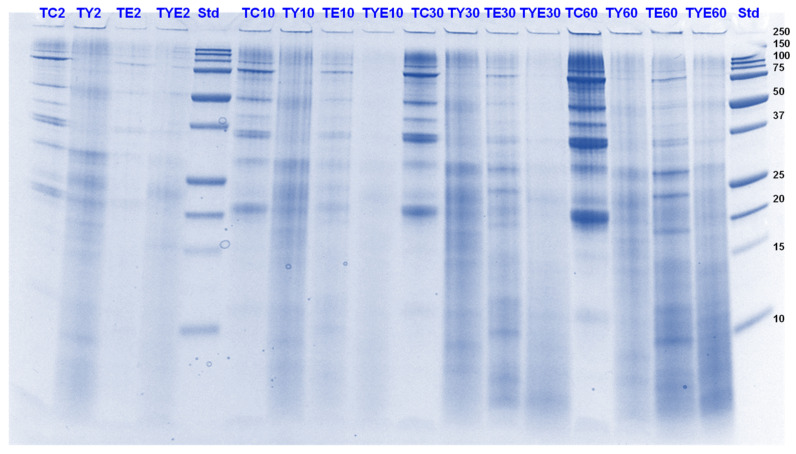
Tricine SDS-PAGE electrophoretogram of tofu (T) digest after 2, 10, 30 and 60 min of simulated oral-gastric digestion in the presence or absence of **SunGold (Y)** kiwifruit extract. TC, Tofu (T) control without any digestive enzymes; TK, with added kiwifruit extract but no digestive enzymes; TE, with digestive enzymes but no added kiwifruit extract; TYE, with both digestive enzymes and gold kiwifruit extract. Numbers 2, 10, 30 and 60 are the gastric digestion time following 2 min of oral digestion. Bands (1–10) in the molecular weight standard lane correspond to molecular weights 250, 150, 100, 75, 50, 37, 25, 20, 15 and 10 kDa.

**Figure 8:**
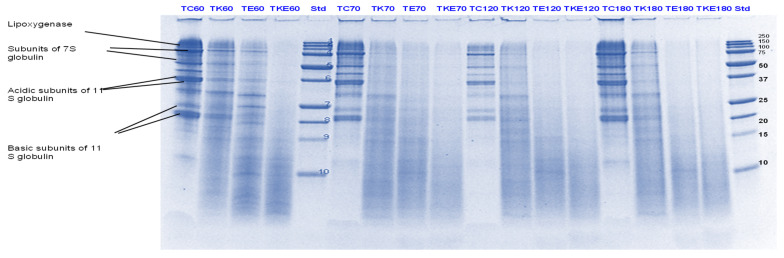
Tricine SDS-PAGE electrophoretogram of tofu (T) digest after 60, 70, 120 and 180 min of simulated oral-gastric-small intestinal digestion in the presence or absence of **Hayward (K)** kiwifruit extract. TC, tofu (T) control without any digestive enzymes; TK, with added green kiwifruit extract but no digestive enzymes; TE, with digestive enzymes but no added kiwifruit extract; TKE, with both digestive enzymes and green kiwifruit extract. Bands (1–10) in the molecular weight standard lane correspond to molecular weights 250, 150, 100, 75, 50, 37, 25, 20, 15 and 10 kDa. The number 60 is the gastric digestion time following 2 min of oral digestion. Numbers 70, 120 and 180 are the total digestion times in oral-gastro-small intestinal digestion (including the first 60 min of gastric digestion).

**Figure 9:**
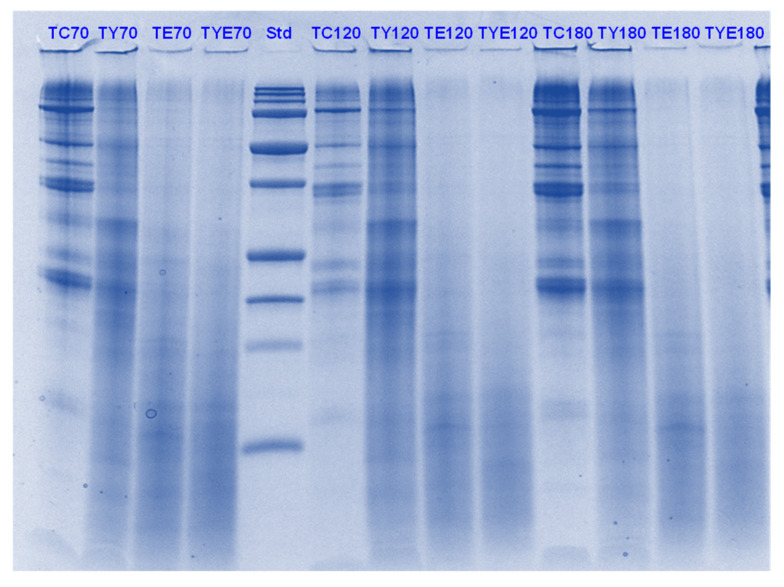
Tricine SDS-PAGE electrophoretogram of tofu (T) digest after 70, 120 and 180 min of simulated oral-gastric-small intestinal digestion in the presence or absence of **SunGold (Y)** kiwifruit extract. TC, tofu (T) control without any digestive enzymes; TY, with added gold kiwifruit extract but no digestive enzymes; TE, with digestive enzymes but no added kiwifruit extract; TYE, with both digestive enzymes and gold kiwifruit extract. Bands (1–10) in the molecular weight standard lane correspond to molecular weights 250, 150, 100, 75, 50, 37, 25, 20, 15 and 10 kDa. Numbers 70, 120 and 180 are the total digestion times in oral-gastro-small intestinal digestion (including the first 60 min of gastric digestion).

Tofu digests were analysed using SDS-PAGE. The electrophoretograms are presented in Figure 6, Figure 7, Figure 8 and Figure 9. The lanes with control protein showed that tofu mainly comprised the soy proteins lipoxygenase (95 kDa), β-conglycinin (7S globulin), and glycinin (11S globulin). The main identified proteins α’, α β-subunits (80, 76, and 50 kDa) of 7S globulin; acidic subunits (38, 35, and 33 kDa) of 11S globulin; and basic subunits (25, 22, and 18 kDa) of 11S globulin [38,39,40].

As observed for PPI, there was significant protein breakdown observed in tofu digests that were incubated in the presence of either Hayward or SunGold kiwifruit extracts alone (with no added pepsin) during the gastric digestion phase; with the former showing better effects on tofu protein digestion. However, the effects of both the extracts were found to be more pronounced when they were present along with pepsin (Figure 6 and Figure 7). This could be observed throughout the gastric phase, with a significant breakdown of most of the parent proteins and the appearance of peptides with MW < 10 kDa in the kiwifruit extract containing digests. 

The rate of protein breakdown during the small intestinal digestion phase and overall digestion (after 180 min of digestion) of tofu proteins seemed to be enhanced by kiwifruit extract when present along with digestive enzymes, with the kiwifruit extract containing digests showing greater protein and peptide breakdown than the digestive enzymes containing digests (Figure 8 and Figure 9). No large differences could be observed among Hayward or SunGold kiwifruit containing digests at the end of the digestion when added along with pepsin, suggesting a significant role of SunGold kiwifruit in soy protein digestion despite having lower actinidin activity. This leads to the conclusion that the actinidin activity of SunGold is enough to open up protein structure for achieving better overall protein hydrolysis, but the rate of protein hydrolysis of protein can be improved by higher actinidin levels, similar to those present in the green kiwifruit.

Green kiwifruit extract when present alone led to better hydrolysis of proteins both during gastric and small-intestinal digestion when compared to SunGold kiwifruit (alone, with no added digestive enzymes throughout the digestion), with its effect being more pronounced in the gastric digestion phase, which was expected due to lower actinidin activity of the latter. The observed are similar to those observed for PPI in terms of pronounced effects of kiwifruit extracts observed during the gastric digestion phase, however, the soy proteins in tofu were degraded more quickly than pea proteins. The enhancement in the rate of proteolysis during the gastric digestion phase by actinidin led to enhancement in overall hydrolysis of the tofu proteins and peptides by the digestive enzymes, both in the gastric and in the small intestinal digestion phase.

#### 3.3.2. Free Amino N (%) and Soluble Nitrogen (%)

**Figure 10:**
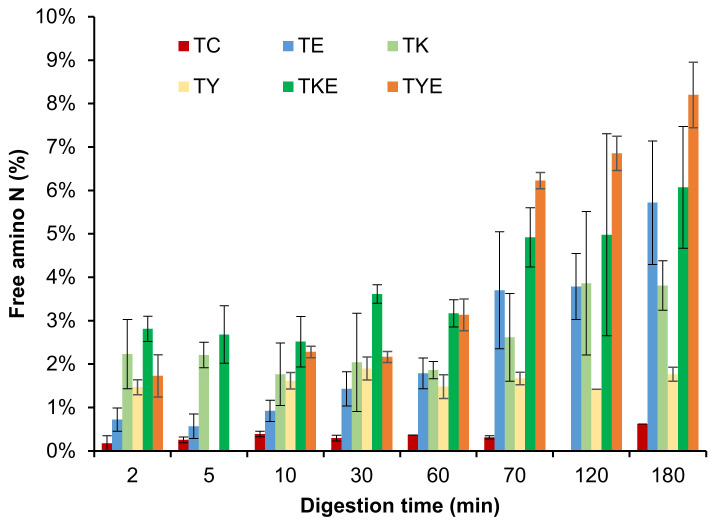
Free amino N (%) release during simulated gastro-small intestinal digestion of tofu in the presence or absence of Hayward or SunGold kiwifruit extract. TC, tofu (T) control without any digestive enzymes; TK/TY, with added green/gold kiwifruit extract but no digestive enzymes; AE, with digestive enzymes but no added kiwifruit extract; TKE/TYE, with both digestive enzymes and green/gold kiwifruit extract. Numbers 2, 10, 30 or 60 is the gastric digestion time following 2 min of oral digestion. Numbers 70, 120 or 180 are the total digestion times in oral-gastro-small intestinal digestion (including the first 60 min of gastric digestion).

Free amino N release and protein solubility throughout digestion was observed to be significantly greater for kiwifruit extract-containing digests than for the control protein digests incubated in buffers alone (in the absence of digestive enzymes) (Figure 10). Greater effects of green kiwifruit extract on free amino N release agree well with the SDS-PAGE results. Furthermore, no significant differences (*p* < 0.05) could be observed for the free amino N release among the digestive enzymes containing tofu digests and the green kiwifruit extract containing sample (with no added digestive enzymes), showing the potential of actinidin from both Hayward and SunGold kiwifruits in protein digestion, particularly for people with an impaired digestive system.

There was considerably higher free amino N released during the first five minutes of gastric digestion in the digests containing kiwifruit extract (gold or Hayward) compared to the digests containing digestive enzymes alone. This suggests that actinidin present in kiwifruit extract helps to break down proteins to a greater extent than digestive enzymes alone, leading to faster peptide release.

### 3.4. Almonds

#### 3.4.1. SDS-PAGE

Pru du 6, also known as amandin or prunin, accounts for approximately 70% of the total soluble protein in almonds [41]. Along with being the major almond protein component, it is also known to be its major allergen [42,43]. Prunin is composed of two polypeptides: pruning-1 (Pru-1, MW 61.0 kDa) and pruning-2 (Pru-2, MW 55.9 kDa), assembled through disulfide linkages. Pru-1 is composed of an acidic α-chain of 40.1 kDa and a basic β-chain of 20.9 kDa, whereas Pru-2 has two subunits of 34.5 kDa (α-chain) and 21.4 kDa (β-chain), corresponding to the α- and β-chains, respectively [44].

**Figure 11:**
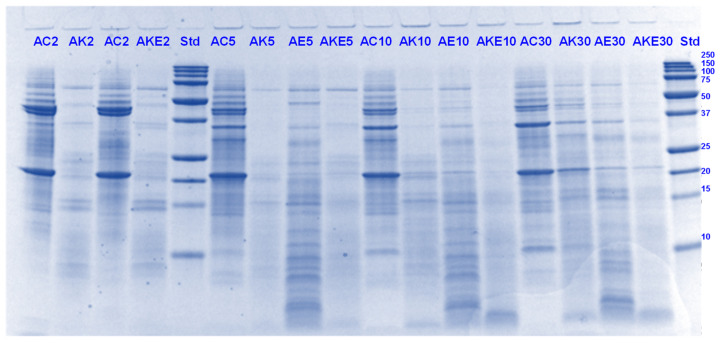
Tricine SDS-PAGE electrophoretogram of almond (A) digests after 2, 5, 10, and 30 min of simulated oral gastric digestion in the presence or absence of **Hayward (K)** kiwifruit extract. AC, almond (A) control without any digestive enzymes; AK, with added green kiwifruit extract but no digestive enzymes; AE, with digestive enzymes but no added kiwifruit extract; AKE, with both digestive enzymes and green kiwifruit extract. Numbers 2, 5, 10, and 30 are gastric digestion times following 2 min of oral digestion. Bands (1–10) in the molecular weight standard lane correspond to molecular weights 250, 150, 100, 75, 50, 37, 25, 20, 15 and 10 kDa.

**Figure 12:**
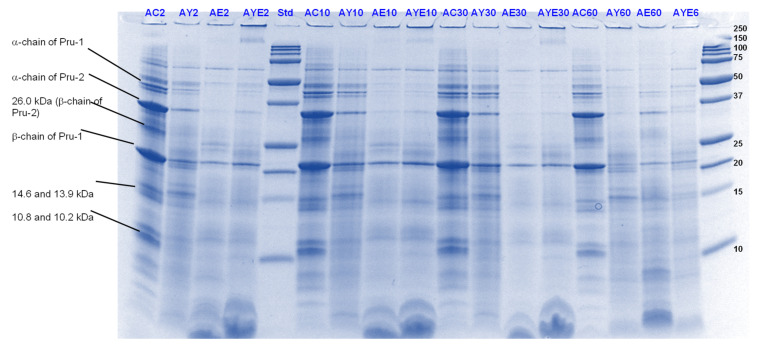
Tricine SDS-PAGE electrophoretogram of almond (A) digests after 2, 10, 30, and 60 min of simulated oral gastric digestion in the presence or absence of **SunGold (Y)** kiwifruit extract. AC, almond (A) control without any digestive enzymes; AY, with added gold kiwifruit extract but no digestive enzymes; AE, with digestive enzymes but no added kiwifruit extract; AYE, with both digestive enzymes and gold kiwifruit extract. Numbers 2, 10, 30 and 60 are gastric digestion times following 2 min of oral digestion. Bands (1–10) in the molecular weight standard lane correspond to molecular weights 250, 150, 100, 75, 50, 37, 25, 20, 15 and 10 kDa.

Under the present reducing conditions, released acid and basic subunits of Pru-1 and Pru-2 can be identified in the lanes containing the control (with no added digestive enzymes) almond protein digests (Figure 11 and Figure 12). Bands corresponding to molecular weights about 14 and 10 kDa could be attributed to Pru du 4 and Pru du 5 [41]. Upon the addition of Hayward kiwifruit extract, a general decrease in band intensity was displayed, throughout the gastric phase with concomitant disappearance of many signals corresponding to Pru-1 and Pru-2 acidic and basic chains that were observed in the control protein lane. As observed for PPI, throughout the gastric phase, Hayward kiwifruit extract, whether alone or in the presence of digestive enzymes, enhanced the breakdown of most of the almond proteins and peptides over the sample that contained digestive enzymes alone. 

On the other hand, the effect of SunGold kiwifruit extract was not that pronounced on the digestion of almond proteins. However, almonds digested in the presence of SunGold extract alone (with no added digestive enzymes) showed considerable protein breakdown, particularly during the gastric digestion phase (Figure 12). 

**Figure 13:**
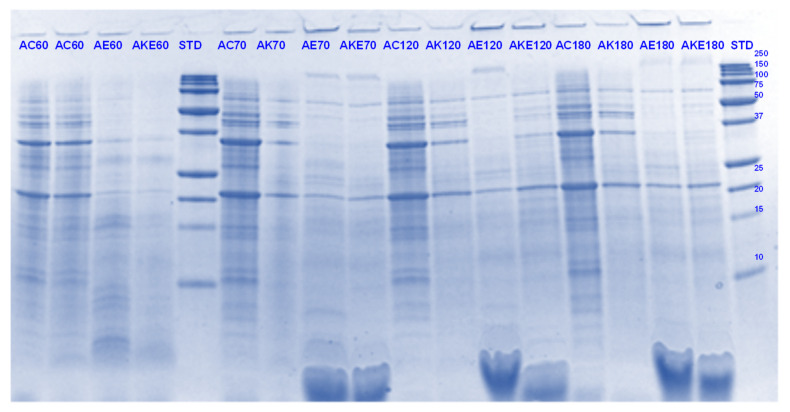
Tricine SDS-PAGE electrophoretogram of almond (A) digests after 60, 70, 120 and 180 min of simulated oral-gastric-small intestinal digestion in the presence or absence of **Hayward (K)** kiwifruit extract. AC, almond (A) control without any digestive enzymes; AK, with added green kiwifruit extract but no digestive enzymes; AE, with digestive enzymes but no added kiwifruit extract; AKE, with both digestive enzymes and green kiwifruit extract. The number 60 is the gastric digestion time following 2 min of oral digestion. Numbers 70, 120 and 180 are the total digestion times in oral-gastro-small intestinal digestion (including 60 min of gastric digestion). Bands (1–10) in the molecular weight standard lane correspond to molecular weights 250, 150, 100, 75, 50, 37, 25, 20, 15 and 10 kDa.

**Figure 14:**
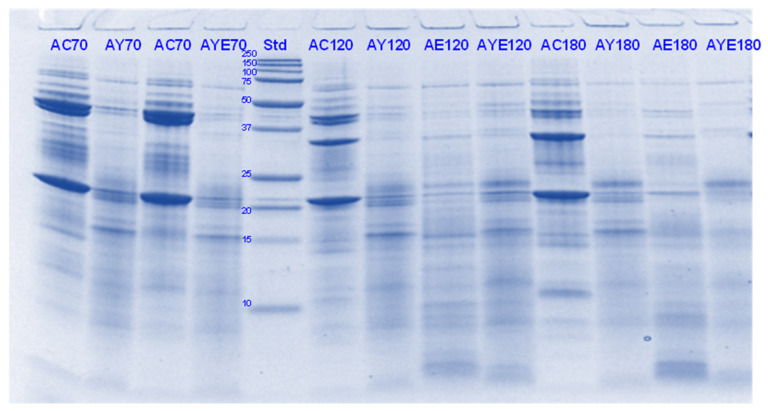
Tricine SDS-PAGE electrophoretogram of almond (A) digests after 70, 120 and 180 min of simulated oral-gastric-small intestinal digestion in the presence or absence of **SunGold (Y)** kiwifruit extract. AC, almond (A) control without any digestive enzymes; AY, with added gold kiwifruit extract but no digestive enzymes; AE, with digestive enzymes but no added kiwifruit extract; AYE, with both digestive enzymes and gold kiwifruit extract. Numbers 70, 120 and 180 are the total digestion times in oral-gastro-small intestinal digestion (including 60 min of gastric digestion). Bands (1–10) in the molecular weight standard lane correspond to molecular weights 250, 150, 100, 75, 50, 37, 25, 20, 15 and 10 kDa.

During the small-intestinal phase, AKE (with both digestive enzymes and Hayward kiwifruit extract) digests showed better protein breakdown than AK (Hayward kiwifruit only) digests. However, not much change was observed in protein or peptide breakdown between the former and the digests containing digestive enzymes alone (Figure 13 and Figure 14). The peptide profiles were also observed to differ among the SunGold and green kiwifruit extract-containing digests, particularly during the small intestinal digestion phase. Compared with the legume proteins tested, actinidin (alone, with no digestive enzymes) seemed to be contributing to the breakdown of proteins and peptides during the small-intestinal digestion phase too as evident from the differences in the intensities of the major bands among 60 min of gastric digestion and the small-intestinal protein digestion.

#### 3.4.2. Free Amino N (%) and Soluble Nitrogen (%)

**Figure 15:**
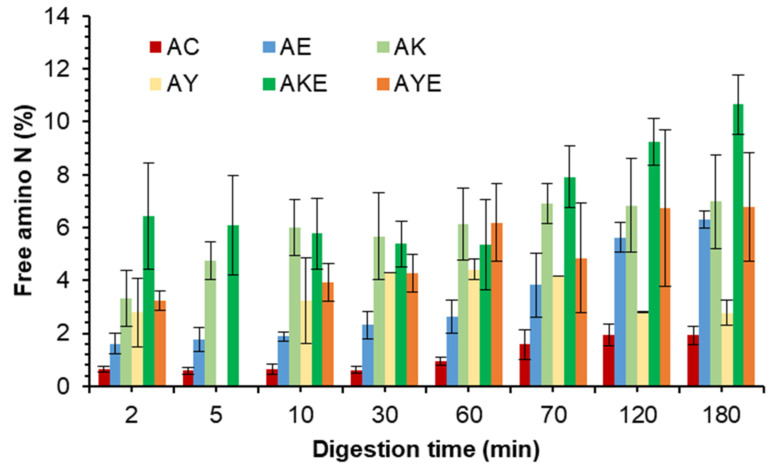
Free amino N (%) release during simulated gastro-small intestinal digestion of almonds in the presence or absence of Hayward or SunGold kiwifruit extract. AC, almond (A) control without any digestive enzymes; AK/AY, with added green/gold kiwifruit extract but no digestive enzymes; AE, with digestive enzymes but no added kiwifruit extract; AKE/AYE, with both digestive enzymes and green/gold kiwifruit extract. Numbers 2, 10, 30 and 60 are the gastric digestion time following 2 min of oral digestion. Numbers 70, 120 and 180 are the total digestion times in oral-gastro-small intestinal digestion (including the first 60 min of gastric digestion).

The results for free amino N release are consistent with those reported in Section 3.4.1 for protein breakdown (Figure 15). Digests containing both Hayward kiwifruit extract and digestive enzymes displayed enhanced free amino N release throughout the simulated digestion compared to the digests containing digestive enzymes alone. 

SunGold kiwifruit (when added along with digestive enzymes) also led to an enhancement in both protein solubility (data not shown) and free amino N release but during the gastric phase alone. No positive effect of SunGold kiwifruit could be observed on free amino N release or protein solubility during the small intestinal phase.

Almond protein has been reported to be of poor nutritional quality in terms of its Protein Digestibility Corrected Amino Acid Scoring (PDCAAS) [45]. In this study, it was noted that although almond protein breakdown (disappearance of proteins and peptides with MW >10 kDa, observed through SDS-PAGE) was slower and lower than both legume proteins tested, the values of free amino N released were higher than both soy and pea proteins, particularly for the kiwifruit extract containing samples and for the gastric digestion phase. The higher and faster release of free amino N compared to other sources of proteins when acted upon by actinidin present in green or gold kiwifruit could be due to its higher specificity for almond proteins and peptides.

### 3.5. Quinoa

Quinoa has been selected by FAO as one of the crops with the potential to offer food security due to its greater tolerance to salinity and drought stress [46,47]. Besides having a high protein content, Quinoa seeds have a balanced essential amino acid content, with higher lysine and methionine contents than traditional cereals such as rice, maize, barley and wheat. The quality of quinoa protein is comparable to that of casein from milk [48].

#### 3.5.1. SDS-PAGE

**Figure 16:**
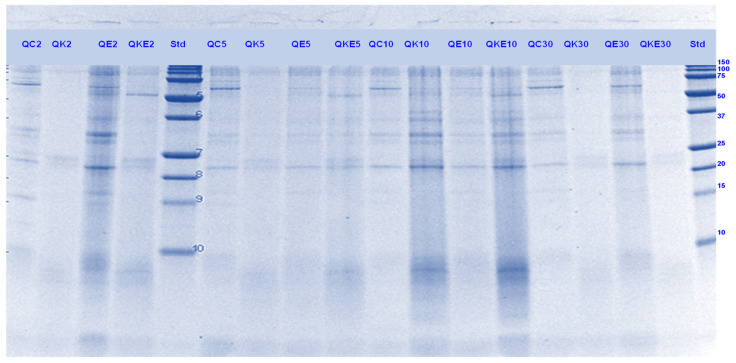
Tricine SDS-PAGE electrophoretogram of quinoa (Q) digests after 2, 5, 10, and 30 min of simulated oral-gastric digestion in the presence or absence of **Hayward (K)** kiwifruit extract. QC, Quinoa (Q) control without any digestive enzymes; QK, with added green kiwifruit extract but no digestive enzymes; QE, with digestive enzymes but no added kiwifruit extract; QKE, with both digestive enzymes and green kiwifruit extract. Numbers 2, 5, 10, and 30 are the gastric digestion times following 2 min of oral digestion. Bands (1–10) in the molecular weight standard lane correspond to molecular weights 250, 150, 100, 75, 50, 37, 25, 20, 15 and 10 kDa.

**Figure 17:**
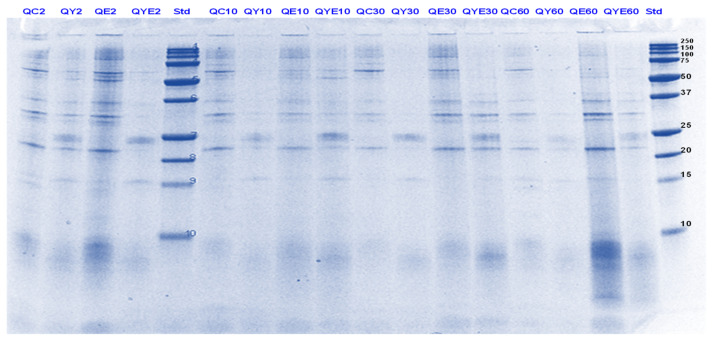
Tricine SDS-PAGE electrophoretogram of quinoa (Q) digests after 2, 10, 30 and 60 min of simulated oral-gastric digestion in the presence or absence of **SunGold (Y)** kiwifruit extract. QC, Quinoa (Q) control without any digestive enzymes; QY, with added gold kiwifruit extract but no digestive enzymes; QE, with digestive enzymes but no added kiwifruit extract; QKY, with both digestive enzymes and gold kiwifruit extract. Numbers 2, 10, 30 and 60 are the gastric digestion time following 2 min of oral digestion. Bands (1–10) in the molecular weight standard lane correspond to molecular weights 250, 150, 100, 75, 50, 37, 25, 20, 15 and 10 kDa.

**Figure 18:**
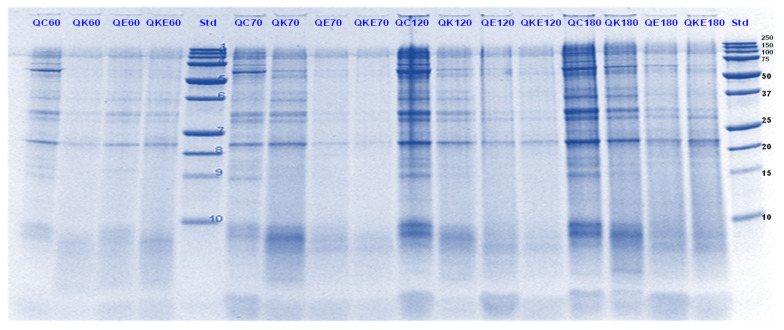
Tricin SDSPAGE electrophoretogram of quinoa (Q) digests after 60, 70, 120 and 180 min of simulated oral-gastric-small intestinal digestion in the presence or absence of **Hayward (K)** kiwifruit extract. QC, Quinoa (Q) control without any digestive enzymes; QK, with added green kiwifruit extract but no digestive enzymes; QE, with digestive enzymes but no added kiwifruit extract; QKE, with both digestive enzymes and green kiwifruit extract. The number 60 is the gastric digestion time following 2 min of oral digestion. Numbers 70, 120 and 180 are the total digestion times in oral-gastro-small intestinal digestion (including 60 min of gastric digestion). Bands (1–10) in the molecular weight standard lane correspond to molecular weights 250, 150, 100, 75, 50, 37, 25, 20, 15 and 10 kDa.

**Figure 19:**
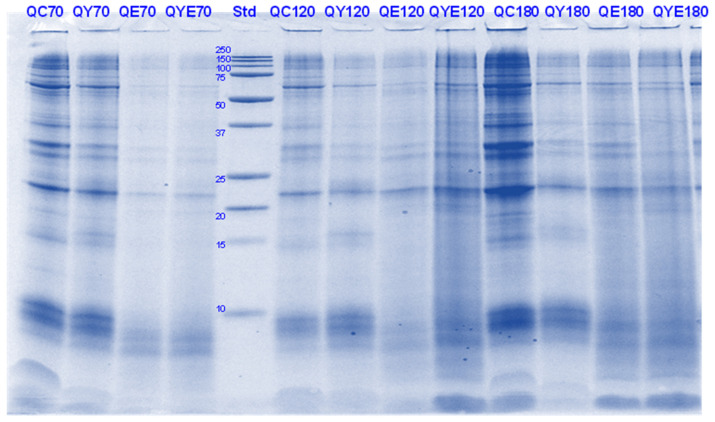
Tricine-SDS-PAGE electrophoretogram of quinoa (Q) digests after 70, 120 and 180 min of simulated oral-gastric-small intestinal digestion in the presence or absence of **SunGold (Y)** kiwifruit extract. QC, Quinoa (Q) control without any digestive enzymes; QY, with added gold kiwifruit extract but no digestive enzymes; QE, with digestive enzymes but no added kiwifruit extract; QKY, with both digestive enzymes and gold kiwifruit extract. Numbers 70, 120 and 180 are the total digestion times in oral-gastro-small intestinal digestion (including 60 min of gastric digestion). Bands (top to bottom) in the molecular weight standard lane (Std) correspond to molecular weights 250, 150, 100, 75, 50, 37, 25, 20, 15 and 10 kDa.

Acidic or α- (MW ~28 and 35 kDa) and basic or β- (MW ~18 and 22 kDa) subunits of chenopodin (11 S type globulin), which is the main protein in quinoa seeds were identified in the gel electrophoretogram ([46]; Figure 16, Figure 17, Figure 18 and Figure 19). Other identified components were the chenoprotein (31 kDa; [49]) and low molecular weight albumin components (<20 kDa; [46]). 

Quinoa proteins were less soluble during the gastric digestion phase than during the small-intestinal conditions. Overall, there was a greater protein breakdown observed for the samples digested with Hayward kiwifruit extract alone (in the absence of any digestive enzymes, Figure 16 and Figure 17) during the simulated gastric phase. For the sample that contained both Hayward kiwifruit extract and digestive enzymes, the effects on protein breakdown were also considerably positive for digestion during the early stomach phase and also during the initial stages of the small-intestinal phase, compared with the sample digested using digestive enzymes alone.

For the SunGold kiwifruit extract-containing samples, enhanced protein breakdown was observed, particularly during the gastric phase. Thereafter no positive effect of KE could be observed. The bands with MW 31, 28 and 22 kDa along with some high (>50 kDa) MW bands, which were present in the control protein digests were observed in all the enzyme/kiwifruit extract containing digests at the end of digestion, suggesting their resistance towards enzyme action (Figure 18 and Figure 19)

Cooked quinoa proteins were found to be least digestible as observed through SDS-PAGE compared to the other proteins analysed in this study. The protein digestibility of quinoa has been reported to decrease with heating while soy and pea proteins become more digestible, depending on the heating temperature [50]. The other components present in quinoa including starch and fibre might have affected its digestibility too [36].

#### 3.5.2. Free Amino N (%) and Soluble Nitrogen (%)

**Figure 20:**
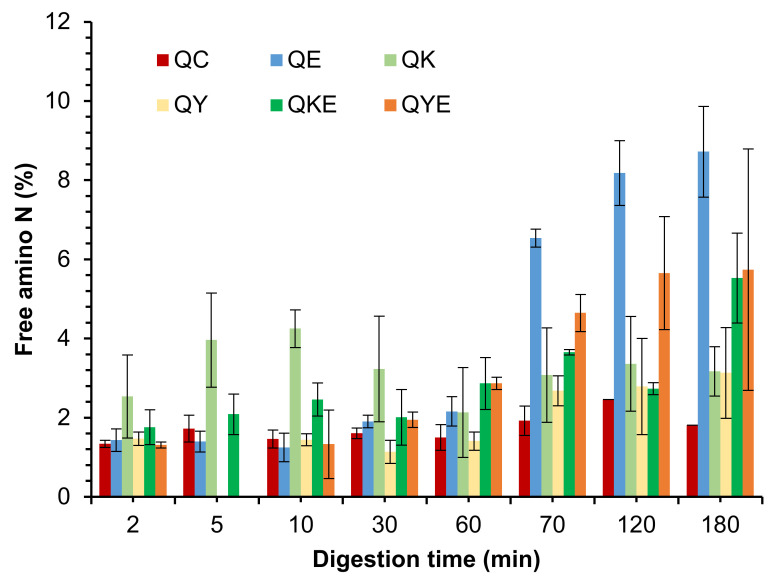
Free amino N (%) release during simulated gastro-small intestinal digestion of quinoa in the presence or absence of Hayward or SunGold kiwifruit extract. QC, Quinoa (Q) control without any digestive enzymes; QK/AY, with added green/gold kiwifruit extract but no digestive enzymes; QE, with digestive enzymes but no added kiwifruit extract; QKE/QKY, with both digestive enzymes and green/gold kiwifruit extract. Numbers 2, 10, 30 and 60 are the gastric digestion time following 2 min of oral digestion. Numbers 70, 120 or 180 are the total digestion times in oral-gastro-small intestinal digestion (including the first 60 min of gastric digestion).

Despite seeing enhanced protein breakdown for the green kiwifruit extract-containing samples, no positive effects of the addition of kiwifruit extract could be observed on free amino N release (Figure 20) or protein solubility (data not shown) during simulated gastro-small intestinal digestion. Some positive effects were observed only for Hayward kiwifruit extract (no added digestive enzymes) after 10 min of gastric digestion, which is well supported by the SDS-PAGE results that showed the highest protein breakdown in those digests. 

## 4. Conclusions

The effects of both SunGold and Hayward kiwifruit extracts in terms of protein breakdown (through SDS-PAGE) and free amino N (ninhydrin) release during digestion of a range of plant proteins were studied. Important increases in the rates of gastric digestion in the presence of actinidin (kiwifruit extract from Hayward fruit) were observed for PPI, almond and tofu, with small effects seen with quinoa proteins. The SunGold variety has only about one-quarter of the actinidin activity of that found in Hayward fruit. This is nevertheless a significant amount of proteolytic activity and was found to have significant positive effects on the digestion of many food proteins mentioned above. Incubation in the presence of kiwifruit extract alone led to significant protein and peptide breakdown for all the foods, which is a useful result for people with a compromised digestive system. In some cases, e.g., in the case of almonds, the effect of SunGold kiwifruit extract alone (no added digestive enzymes) on protein breakdown was even greater than when both kiwifruit extract and digestive enzymes were present. This could be due to the hydrolysis of proteases (actinidin) present in the kiwifruit extract by the digestive proteases, leading to decreased overall food protein breakdown. Another interesting observation was that the SunGold extract alone had a slightly greater effect on protein breakdown than the green kiwifruit extract alone during the small intestinal digestion phase possibly due to differences in the composition of the extracts.

The disappearance of many signals corresponding to Pru-1 and Pru-2 acidic and basic chains of Pru-du 6, which are known to be the major allergens present in almonds, was observed through SDS-PAGE. Further studies are required to investigate if kiwifruit has any role to play in reducing almond protein allergenicity.

The outcome of this work suggests potential digestive and other health benefits may be achieved through the consumption of both green and SunGold kiwifruit. The amount of extract obtained from SunGold was considerably higher than that from Hayward, possibly due to SunGold’s lower total and insoluble fibre and higher water contents. The differences in the composition of the extracts could be responsible for the differences in their observed effects on protein digestion. We also assume that actinidin release from SunGold microstructure would be much easier and quicker following consumption due to microstructural differences among SunGold and Hayward fruits. This may further impact protein digestion kinetics and warrants further investigation. The analysis of the peptides released during digestion will be investigated in the future using mass spectrometry to understand the mechanisms of protein breakdown by actinidin. It is important to mention that the present results are derived from a model system and, although a useful predictor of what will happen in vivo, need to be further validated in animal and/or clinical trials.

## Figures and Tables

**Table 1:** Nitrogen, crude protein contents ^1^ of foods; kiwifruit extract enzyme activities ^2^ and other information.

Food	Protein Content (%) ^3^	Average Enzyme Activity (U/mL of Extract or U/g of Fresh Fruit)	Serve Size (g)
Tofu	12.46 ± 0.05 ^b^	--	100
Almonds	20.36 ± 0.21 ^a^	--	30
Cooked Quinoa	3.34 ± 0.07 ^d^	--	185
Pea protein isolate (paste)	11.38 ± 0.04 ^c^	--	100
Green (Hayward) kiwifruit extract	0.37 ± 0.00 ^e^	27.9 U/mL or ~8.4 U/g of fresh fruit	60 (based on 2 fruits, 112 g average fruit size)
SunGold kiwifruit extract	0.40 ± 0.00 ^e^	7.8 U/mL or ~3.9 U/g of fresh fruit	100 (based on 2 fruits, 114 g average fruit size)

^1^ Determined using the AOAC Kjeldahl method; ^2^ determined using the method described by [4,5] Kaur et al. (2010a) ^3^ Nitrogen to protein conversion factors: 6.25 for tofu and quinoa; 5.18 for almonds; 5.4 for pea protein isolate; 5.64 for kiwifruit extracts. ^a–e^ Means within a column with the same superscript letter are not significantly different (*p* < 0.05).
